# JNK Activation in Alzheimer’s Disease Is Driven by Amyloid β and Is Associated with Tau Pathology

**DOI:** 10.1021/acschemneuro.3c00093

**Published:** 2023-03-28

**Authors:** Maite Solas, Silvia Vela, Cristian Smerdou, Eva Martisova, Iván Martínez-Valbuena, María-Rosario Luquin, María J. Ramírez

**Affiliations:** † Department of Pharmacology and Toxicology, University of Navarra, 31008 Pamplona, Spain; ‡ IdISNA, Navarra Institute for Health Research, 31008 Pamplona, Spain; § Division of Gene Therapy and Regulation of Gene Expression, Cima Universidad de Navarra, 31008 Pamplona, Spain; ∥ Neurosciences Division, Cima Universidad de Navarra, 31008 Pamplona, Spain; ⊥ Tanz Centre for Research in Neurodegenerative Diseases, University of Toronto, Toronto, Ontario M5S 1A8, Canada; # Neurology Department, Clinica Universidad de Navarra, 31008 Pamplona, Spain

**Keywords:** beta-amyloid, Tau, cognition, hippocampus, neuroinflammation

## Abstract

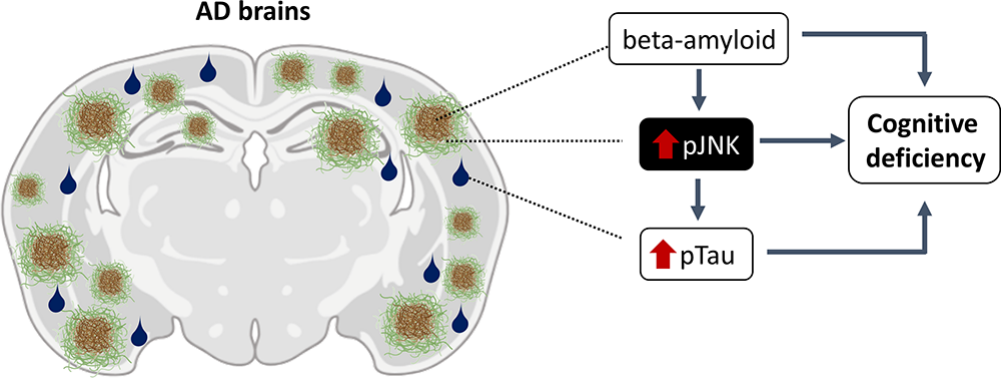

c-Jun N-terminal kinase 3 (JNK3) is suggested to play a key role in neurodegenerative disorders, especially in Alzheimer’s disease (AD). However, it remains unclear whether JNK or amyloid β (Aβ) appears first in the disease onset. Postmortem brain tissues from four dementia subtypes of patients (frontotemporal dementia, Lewy body dementia, vascular dementia, and AD) were used to measure activated JNK (pJNK) and Aβ levels. pJNK expression is significantly increased in AD; however, similar pJNK expression was found in other dementias. Furthermore, there was a significant correlation, co-localization, and direct interaction between pJNK expression and Aβ levels in AD. Significant increased levels of pJNK were also found in Tg2576 mice, a model of AD. In this line, Aβ_42_ intracerebroventricular injection in wild-type mice was able to induce a significant elevation of pJNK levels. JNK3 overexpression, achieved by intrahippocampal injection of an adeno-associated viral vector expressing this protein, was enough to induce cognitive deficiencies and precipitate Tau aberrant misfolding in Tg2576 mice without accelerating amyloid pathology. JNK3 overexpression may therefore be triggered by increased Aβ. The latter, together with subsequent involvement of Tau pathology, may be underlying cognitive alterations in early stages of AD.

## Introduction

Alzheimer’s disease (AD) is a neurodegenerative disorder clinically characterized by a progressive cognitive decline that leads to dementia.^1^ Pathologically, AD is defined by extracellular senile plaques composed of amyloid β (Aβ) and intracellular aggregation of abnormally hyperphosphorylated Tau protein.^1,2^

It has been proposed that Aβ accumulations are able to directly induce synaptic dysfunction, enhancement of oxidative stress, and activation of neuroinflammatory cascade.^3,4^ In this scenario, proinflammatory mechanisms have been described to promote activation of diverse intracellular kinases involved in neuroapoptosis and neuronal loss. Among these, c-Jun N-terminal kinase (JNK) has been described to play an important role in regulating stress signaling within neurons.^5−8^ JNK is a mitogen-activated protein kinase (MAPK).^9^ Three different isoforms of JNK have been described. Isoforms 1 and 2 are ubiquitously expressed, whereas isoform 3 is mainly expressed in the brain^10^ and seems to be involved in proapoptotic mechanisms.^11^

Due to the strong correlation of senile plaques with neuroinflammatory response,^4,10^ it is possible to speculate about the relationship between the increased Aβ levels in AD and the activation of JNK. Previous studies have shown an increased expression of phosphorylated JNK (pJNK) in human postmortem brain samples from AD patients and a positive co-localization with Aβ.^12^ Furthermore, it has also been described *in vitro* that Aβ peptides are able to induce JNK activation.^13−15^ Therefore, it might be possible that Aβ-induced activation of JNK^16^ could result in neuroinflammation and contribute to neurodegeneration in AD.

In addition to glycogen synthase kinase 3 (GSK3), p38, and ERK, JNK phosphorylates Tau at various sites that are hyperphosphorylated in paired helical filaments.^17,18^ Furthermore, JNK activity is enhanced in AD mouse models, in which JNK is co-localized with phosphorylated Tau.^19,20^ Notably, the JNK peptide inhibitor, D-JNKI-1, decreased Tau phosphorylation and subsequent aggregation.^20^

In this context, where JNK3 seems to be profoundly involved in neurodegeneration, several JNK3 inhibitors have been tested as a potential future treatment for AD.^21−25^

Based on all the above-mentioned data, the aim of the present work is to study the expression of JNK in AD brains compared to other dementing neurodegenerative entities and its relationship with Aβ pathology. Moreover, the consequences on cognitive performance, amyloid burden, and Tau pathology of JNK3 overexpression in a transgenic mouse model were studied to elucidate whether JNK overactivation is a cause or a consequence of Aβ accumulation.

## Results

### Specific Increases in pJNK Levels in AD Brain Samples and Tg2576 Mouse Model

There were significant differences between age at death among the different pathological conditions (one-way ANOVA, *F*_3,42_ = 11.065, *p* < 0.001) (Table S1), being AD cases older than the rest of the groups (*p* < 0.01). Therefore, two subsets of controls were used, one being considered mature controls (age at death = 65.1 ± 3.86, *n* = 10) for the FTD, VaD, and LBD samples and the other group being named old controls (age at death = 77.14 ± 2.77, *n* = 16) for AD samples.

**Figure 1:**
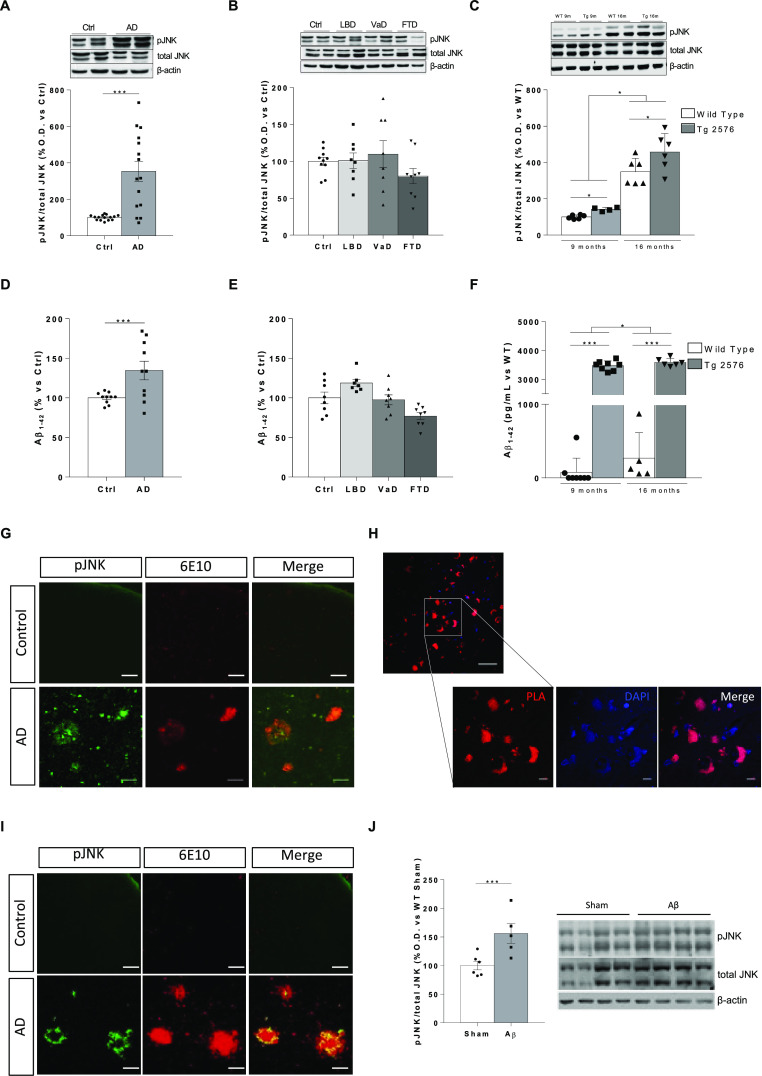
pJNK and Aβ levels in Alzheimer’s disease and other dementias. (A) JNK immunoblotting in control and Alzheimer’s disease (AD) frontal cortex (BA10) (***p < 0.001, Student’s *t*-test) and (B) in vascular dementia (VaD), Lewy body dementia (LBD), and frontotemporal dementia (FTD) (one-way ANOVA, *F*_3,31_ = 1.210, *p* > 0.05). In each panel, a representative picture of western blot is shown. Results are expressed as percent optical density (O.D.) of controls and normalized to total levels of JNK. (C) JNK immunoblotting in 9 and 16 month-old WT and Tg2576 mouse frontal cortices (two-way ANOVA, main effect of genotype, *F*_1,16_ = 5.467, *p* < 0.05; two-way ANOVA, main effect of age, *F*_1,16_ = 84.48, *p* < 0.001). A representative picture of western blot is shown. Results are expressed as percent optical density (O.D.) of WT and normalized to total levels of JNK. (D) Aβ levels in AD cases (*p < 0.05, Student’s *t*-test) and (E) in VaD, LBD, and FTD (one-way ANOVA, *F*_3,36_ = 1.210, *p* > 0.05). Levels of Aβ_42_ are expressed as % versus its corresponding controls (Ctrl). (F) Aβ_42_ levels in 9 and 16 month-old wild-type (WT) and Tg2576 mouse frontal cortices (two-way ANOVA, main effect of genotype, *F*_1,22_ = 181.2, *p* < 0.001; two-way ANOVA, main effect of age, *F*_1,22_ = 12.04, *p* < 0.01). (G) pJNK and 6E10 (β-amyloid marker) immunostaining in BA10 of Alzheimer’s disease (AD) cases. Scale bars, 50 μm. (H) In situ PLA assay for JNK and Aβ in human AD brains. Scale bars, 10 μm. (I) pJNK and 6E10 (β-amyloid marker) immunostaining in the frontal cortex of Tg2576. Scale bars, 50 μm. (J) JNK immunoblotting in wild-type (WT) mouse frontal cortex after ICV administration of Aβ_42_. Results are expressed as percent optical density (O.D.) of sham and normalized to total levels of JNK. ***p < 0.001, Student’s *t*-test.

Significant increases in pJNK levels were seen in the frontal cortex (BA10) of patients with AD compared with controls (Figure 1A). In contrast, pJNK levels were similar in all dementia groups (LBD, FTD, or VaD) compared to control samples (Figure 1B). In parallel with data obtained in human samples, significant increased levels of pJNK were found in 9 and 16 month-old Tg2576 mouse frontal cortices compared to WT animals (Figure 1C). Moreover, pJNK levels significantly increase in the 16 month-old group compared with 9 month-old mice (Figure 1C). Interestingly, an age-dependent pJNK increase was also observed in human samples, as in control subjects, pJNK expression correlated significantly with age (Pearson’s, *r* = 0.563; *p* < 0.05; Figure S1).

### pJNK and Aβ Co-Localize in AD Human Samples and in Tg2576 Mice

In parallel to pJNK expression, Aβ_42_ levels were significantly increased in AD cases compared to controls (Figure 1D) and not in other dementias (Figure 1E). Noteworthy, a significant correlation between enhanced pJNK expression in BA10 and Aβ_42_ levels (Spearman’s rho = 0.733, *p* < 0.05, *n* = 16) in AD was observed. No correlation was found between pJNK expression in BA10 and Aβ_42_ levels in any other type of dementia (Spearman’s rho = 0.143, *p* > 0.05, Spearman’s rho = −0.143, *p* > 0.05, and Spearman’s rho = 0.405, *p* > 0.05, for LBD, FTD, and VaD, respectively).

In the same line, as depicted in Figure 1F, Tg2576 mice showed enhanced amyloid pathology. Moreover, it was observed that this increase follows an age fashion as Aβ_42_ levels are significantly higher in 16 month-old mice with respect to 9 month-old mice (Figure 1F).

Immunohistochemical results revealed that AD patients presented pJNK and senile plaque co-localization (Figure 1G), reinforcing the idea of a strong association between activated JNK and Aβ in AD. *In situ* PLA assay revealed the existence of a direct interaction between JNK and Aβ (Figure 1H).

The same pattern was observed in murine samples, as pJNK in Tg2576 mouse brains was detected around the amyloid plaque, while in matched aged WT mice, pJNK immunolabeling was not seen (Figure 1I). Noteworthily, JNK staining did not co-localize with GFAP (astrocytic marker, Figure S2A) or NeuN (neuronal marker, Figure S2B), suggesting that JNK co-localizes with dystrophic neurites, coinciding with the damage of neuritic processes.

### Aβ42 Intracerebroventricular Administration Increases pJNK Levels in Wild-Type Mice

Our previous data raised the question of whether an increase of Aβ could lead to or be the cause of pJNK elevation. To this end, Aβ_42_ was injected intracerebroventricularly (ICV) in WT mice and pJNK levels were measured. As depicted in Figure 1J, pJNK levels increased in the frontal cortex of WT mice after ICV administration of Aβ_42_.

### Effective JNK3 Overexpression in Tg2576 Mice

**Figure 2:**
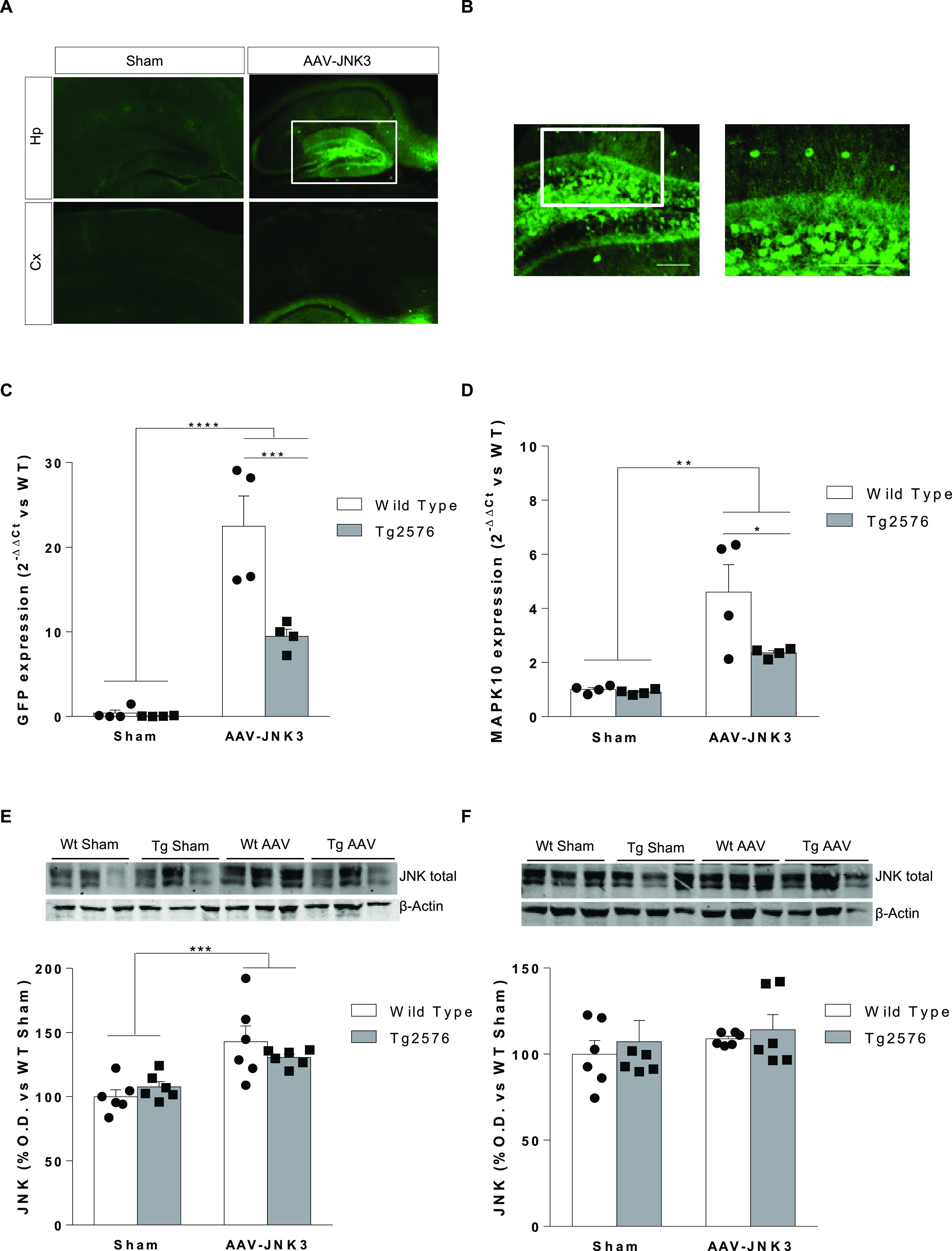
Analysis of the transduction efficacy of the AAV. (A) Cortical and hippocampal slices representative of GFP expression in sham and AAV-JNK3-injected wild-type mice. The white box indicates the key area magnified in panel (B). (B) Magnification of GFP expression in the hippocampus (Hp). Scale bars, 100 μM. (C) GFP mRNA relative expression in Hp (two-way ANOVA, main effect of AAV, *F*_1,12_ = 63.73, *p* < 0.0001; *n* = 5). (D) JNK3 mRNA relative expression in Hp (two-way ANOVA, main effect of AAV, *F*_1,12_ = 24.14, *p* < 0.001; *n* = 5). (E) JNK protein presence in Hp (two-way ANOVA, main effect of AAV, *F*_1,20_ = 21.27, *p* < 0.001; *n* = 6). (F) JNK protein presence in Cx (two-way ANOVA; *n* = 6). Results are shown as the mean ± SEM. In panels (E) and (F), figures show the optical density (O.D.) percentage and an illustrative image of the blotting. Cx: cortex; Hp: hippocampus; O.D.: optical density.

After demonstrating a successful JNK3 expression *in vitro* (Figure S3), a dose of 1 × 10^10^ vp of AAV8-JNK3-GFP vector (AAV group) or PBS (sham group) was injected bilaterally into hippocampal dentate gyrus. In all AAV-JNK3 mice, GFP expression was detected in the injected area, but no fluorescence was observed in the sham group (Figure 2A), showing that a somatic morphology is observed in the injection site (Figure 2B).

qPCR studies showed a significant increase of GFP in the hippocampus of WT and Tg2576 mice (Figure 2C). In a similar way, JNK3 mRNA was markedly increased in the hippocampus of AAV groups (Figure 2D), linked to an accumulation of JNK protein only in the injection site, i.e., hippocampus (Figure 2E), but not in other areas such as frontal cortex (Figure 2F).

### JNK3 Overexpression Induces Cognitive Deficiency

**Figure 3:**
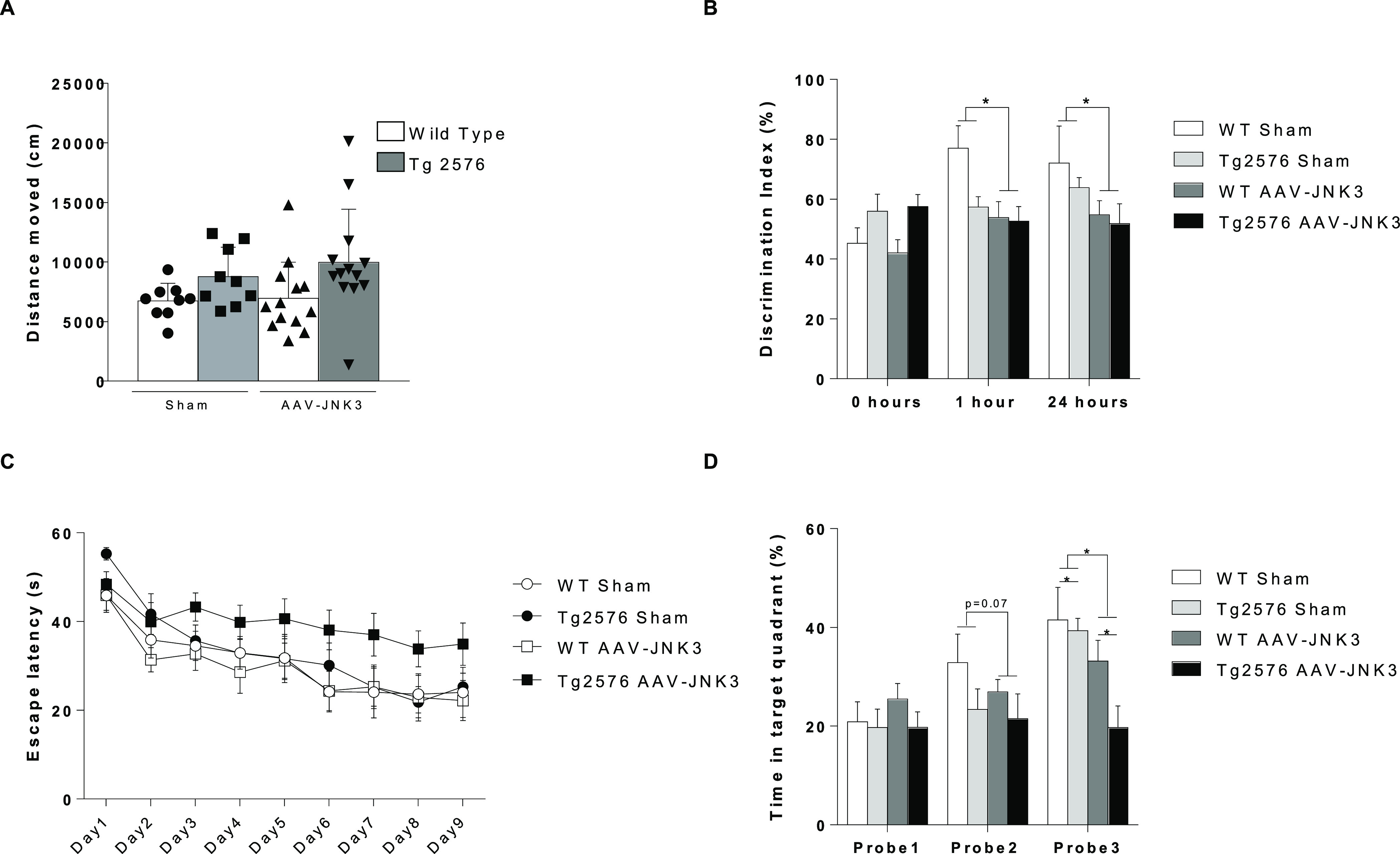
Behavioral consequences of JNK3 overexpression in Hp. (A) Locomotor activity (*n* = 9–12). (B) Cognitive performance in the novel object recognition test (NORT). Data display the discrimination index (time exploring the new object/total exploration time × 100) (1 h task: two-way ANOVA, main effect of AAV, *F*_1,36_ = 6.283, *p* < 0.05; 24 h task: two-way ANOVA, main effect of AAV, *F*_1,31_ = 4.171, *p* < 0.05; *n* = 9–12). Cognitive performance assessed by Morris water maze (MWM). (C) Acquisition phase and (D) retention phase (probe 2: two-way ANOVA, main effect of AAV, *F*_1,37_ = 3.329, *p* = 0.07; probe 3: two-way ANOVA, main effect of AAV, *F*_1,38_ = 15.17, *p* < 0.001 and main effect of genotype, *F*_1,38_ = 6.014, *p* < 0.05) (*n* = 9–12). Data are shown as the mean ± SEM.

No differences were observed in the locomotor activity between groups (Figure 3A). In the NORT (Figure 3B), AAV-JNK3 mice displayed cognitive deficits, as shown by a significantly decreased discrimination index not only in the 1 h task but also in the 24 h test.

In the MWM, as shown in Figure 3C, no significant differences were observed among groups during the invisible-platform phase. Noteworthy, JNK3 overexpression induced cognitive deficiencies in the second and third probe trials not only in Tg2576 mice but also in wild-type mice (Figure 3D).

### Effect of JNK3 Overexpression on Aβ Aggregation

**Figure 4:**
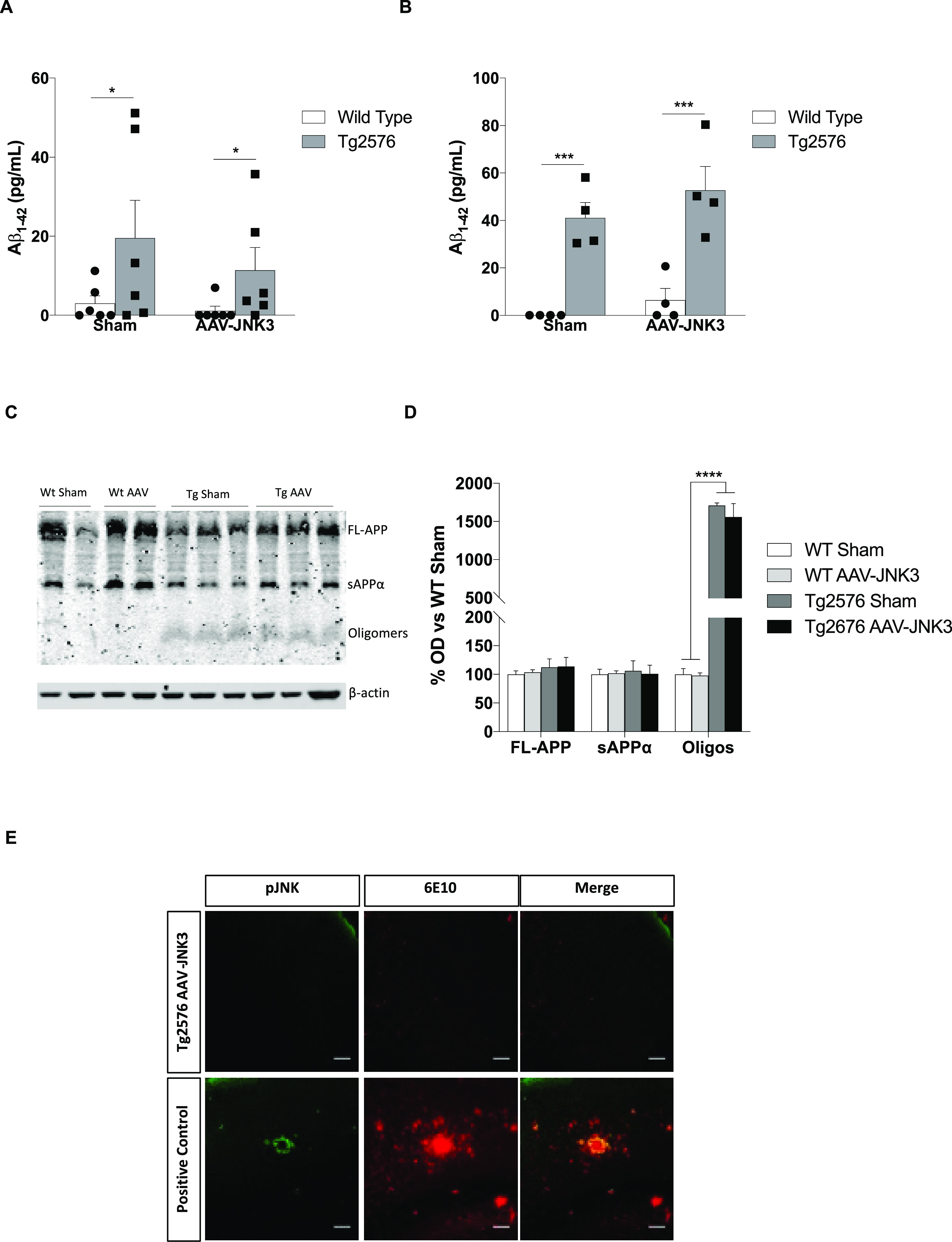
Effect of JNK3 overexpression on Aβ burden. Aβ_42_ levels in 9 month-old Tg2576 mouse hippocampus (A) (two-way ANOVA, main effect of genotype, *F*_1,20_ = 5.531, *p* < 0.05; *n* = 6) and in frontal cortex (B) (two-way ANOVA, main effect of genotype, *F*_1,12_ = 46.20, *p* < 0.001; *n* = 6). (C) Representative Aβ oligomerization immunoblot and (D) quantification (two-way ANOVA, main effect of genotype, *F*_1,9_ = 93.46, *p* < 0.0001; *n* = 4). Results are expressed as the percent optical density (O.D.) of WT sham. (E) pJNK and 6E10 (β-amyloid marker) immunostaining in the frontal cortex of 9 month-old Tg2576 mice vs a positive control (16 month-old Tg2576 mice). Scale bars, 50 μm. O.D.: optical density.

JNK3 was not able to increase total Aβ_1–42_ levels, neither in the hippocampus (Figure 4A) nor in the cortex (Figure 4B). In the same line, JNK3 overexpression was not enough to induce Aβ oligomerization (Figure 4C,D). Furthermore, AAV-JNK3 injection did not precipitate Aβ senile plaque deposition as no 6E10 immunostaining was observed in 9 month-old (3 months post injection) mice compared to a 16 month-old positive control (Figure 4E).

### Effect of JNK3 Overexpression on Tau

**Figure 5:**
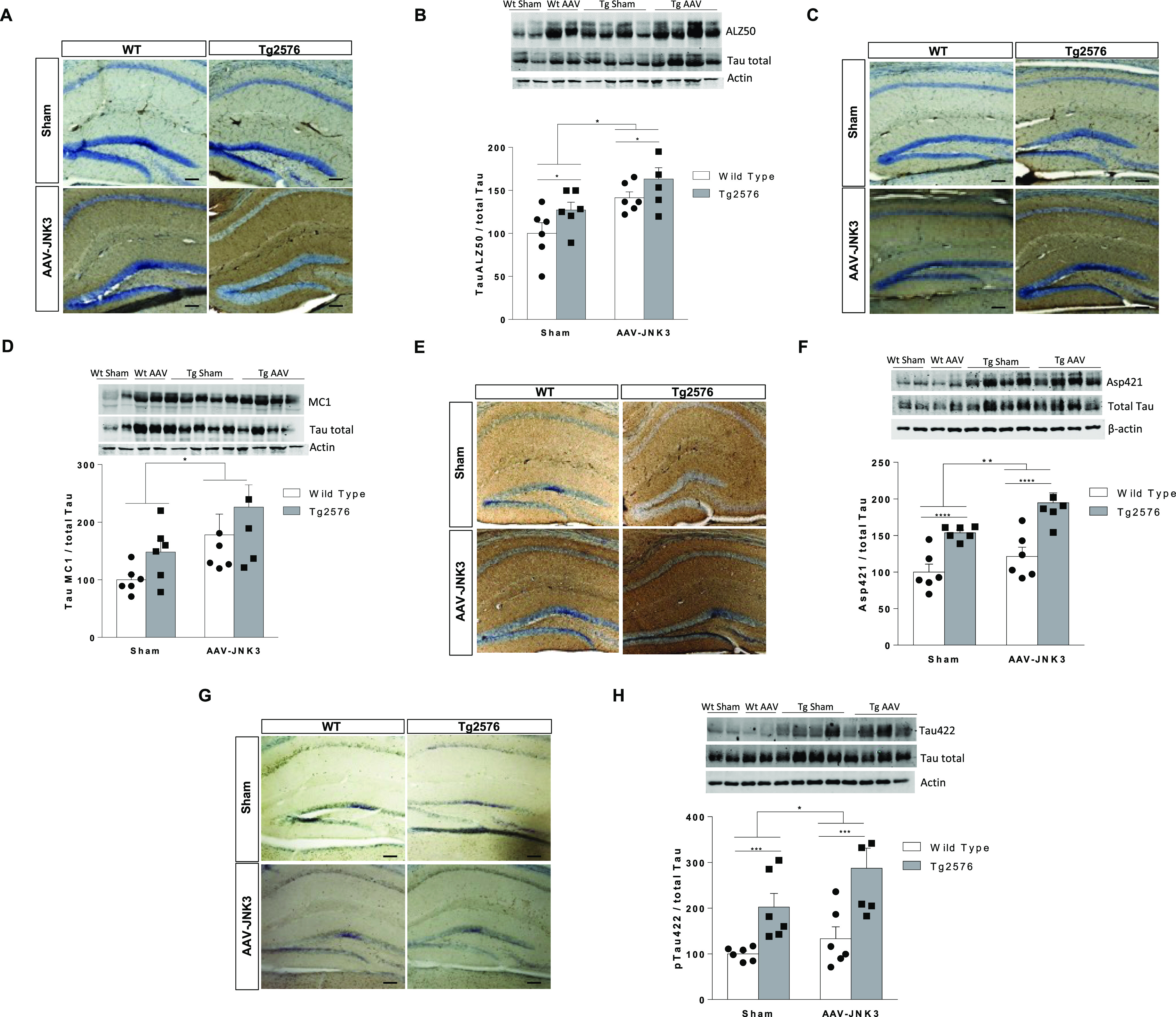
Effect of JNK3 overexpression on Tau pathology. (A) Tau ALZ50 expression analyzed by IHC in sham and AAV-JNK3-injected WT and Tg2576 mice. Scale bars, 100 μM. (B) Tau ALZ50 protein in Hp analyzed by western blotting (two-way ANOVA, main effect of AAV, *F*_1,20_ = 13.07, *p* < 0.001, and main effect of genotype, *F*_1,20_ = 5.223, *p* < 0.05; *n* = 6). (C) Tau MC1 expression analyzed by IHC in sham and AAV-JNK3-injected wild-type and Tg2576 mice. Scale bars, 100 μM. (D) Tau MC1 protein analyzed by western blotting in Hp (two-way ANOVA, main effect of AAV, *F*_1,20_ = 7356, *p* < 0.05; *n* = 6). (E) Asp421-truncated Tau expression analyzed by IHC in sham and AAV-JNK3-injected wild-type and Tg2576 mice. Scale bars, 100 μM. (F) Asp421-truncated Tau protein analyzed by western blotting in Hp (two-way ANOVA, main effect of AAV, *F*_1,20_ = 8.353, *p* < 0.001, and main effect of genotype, *F*_1,20_ = 34.49, *p* < 0.0001; *n* = 6). (G) pTau Ser422 expression analyzed by IHC in sham and AAV-JNK3-injected wild-type and Tg2576 mice. Scale bars, 100 μM. (H) pTau Ser422 analyzed by western blotting in Hp (two-way ANOVA, main effect of AAV, *F*_1,20_ = 3.995, *p* < 0.05, and main effect of genotype, *F*_1,20_ = 18.69, *p* < 0.001; *n* = 6). Results are shown as the mean ± SEM. In panels (B), (D), (F), and (H), figures show the optical density (O.D.) percentage and an illustrative image of the blotting.

In the hippocampus, a strong increase in ALZ50 immunoreactivity was found in AAV-JNK3-injected mice, which was significantly stronger in Tg2576 mice (Figure 5A), as well as, when measured by immunoblotting (Figure 5B), in AAV-JNK3 mice. AAV-JNK3-induced elevation was also obtained for the other Tau conformational form, i.e., MC1 (Figure 5C,D). In the same line, truncated Asp421 (Figure 5E,F) and the preceding Ser422 phosphorylation (Figure 5G,H) appeared to be also significantly increased upon JNK3 overexpression and further exacerbated in Tg2576.

## Discussion

Many studies have pointed out the emerging role of JNK in the development of neurodegenerative processes due to its implication in stress-triggered response,^10,26^ apoptosis,^9^ caspase activation,^26,27^ mitochondrial oxidative burst, gene modulation,^10^ or its involvement in Tau phosphorylation.^5,8,17,28^ Moreover, many different molecules and biological mediators associated with markers of neurodegeneration have proved to directly activate the JNK-c-Jun cascade such as cytokines, reactive oxygen intermediates, or Aβ peptides.^4,6,29,30^ Therefore, JNK has been proposed as a promising target in the field of neurodegenerative disorders.^31^

One of the aims of the present work was to study if the activation of JNK is a central feature in AD rather than in other types of dementias, i.e., VaD, LBD, and FTD. Confirming previously published works, we found increased expression of pJNK in human postmortem brain samples from AD patients and a positive correlation with Aβ levels.^11,12^ Interestingly, this increase of pJNK appeared to be specific to AD, as no alteration in this kinase was observed in the other dementias.

Increases in Aβ levels remain a clear pathological mark, albeit unspecific, in the pathological development of AD, which has been clearly related to neuronal stress and subsequent pathological perpetuator.^32^*In vitro* discoveries revealed that pJNK increases after treatment with Aβ in primary cortical and hippocampal cell cultures.^13−15^ AD experimental models have demonstrated that JNK activation is associated with increased levels of senile plaques.^19^ According to these data in the present study, it has been demonstrated that both Aβ and pJNK increase in the familiar AD model Tg2576. Based on the above-mentioned literature and according to our results, it is tempting to speculate that Aβ accumulation could be the cause of elevated pJNK levels observed in those mice.

Supporting the tight and specific relation between pJNK and Aβ in AD, this work showed the co-localization between pJNK and the β-amyloid senile plaque in the BA10 region of AD patients. The strong Aβ–pJNK interaction was further confirmed by PLA studies. Interestingly, immunohistochemistry in the frontal cortex of Tg2576 mice reproduced the co-localization of pJNK and Aβ with a pattern in which pJNK appears to be located around the senile plaque, suggesting a possible role of pJNK in the inflammation surrounding the plaque and cell death that occurs within that area.

Although co-localization and PLA studies demonstrated a close interaction between Aβ and pJNK, this raises an important question of the present study: is JNK activation the cause or the consequence of Aβ accumulation? There is extensive evidence that Aβ induces the activation of JNK in familiar AD mouse models.^19,33,34^ Moreover, it has been described that Aβ_42_ ICV injection induces astroglial and microglial activation and, as a consequence, neuroinflammation and neurocognitive impairment.^32^ In this line, our study reported a significant increase in pJNK levels after Aβ_42_ ICV injection in healthy mice, according to the published literature^35,36^ and suggesting that pJNK activation is the consequence rather than the cause of Aβ accumulation.

After demonstrating that Aβ can induce JNK activation and in an attempt to elucidate whether pJNK could also lead to Aβ production and accumulation, we induced JNK3 overexpression in Tg2576 mice. JNK3 induction was performed at 6 months of age, i.e., when mice still lack cognitive deficiencies and/or senile plaques, and behavioral studies were performed at 9 months of age when mice still should not show behavioral impairment.^37^ Very interestingly, we found that JNK3 overexpression was associated with a behavioral impairment, not only in Tg2576 but also in WT mice. These data suggest that JNK3 induction is enough to induce alterations in cognitive function. Noteworthily, this cognitive deficiency is not related to a higher Aβ burden because our data showed that JNK3 overexpression does not induce Aβ formation, oligomerization, or senile plaque deposition. Hence, these data indicate that pJNK activation does not seem to be the cause of Aβ accumulation.

Apart from its close relation with Aβ, it has been extensively proposed that JNK kinase induces Tau phosphorylation and subsequent neurofibrillary tangle formation. In the present work, two different Tau conformations, i.e., ALZ50 and MC1, were analyzed to study the implication of JNK3 on Tau aberrant misfolding. Alz50 is an IgM class monoclonal antibody that stains the fibrillar pathology (dystrophic neurites, neurofibrillary tangles, and neuropil threads) commonly observed in postmortem histological analysis of the AD brain.^38^ The Tau conformational change, targeted by the MC1 antibody, is one of the earliest detectable events in the brain of AD patients. This aberrant conformation of Tau was shown to be present in a soluble form of the protein and in paired helical filament (PHF) assemblies.^39^ Importantly, the level of MC1 reactivity correlates with the severity and progression of AD.^40^ Tau truncation has also been related to Tau deposition,^41−44^ and some authors consider C-terminal truncation a primary event, leading to the assembly of Tau into fibrils.^41−49^ Tau truncation is frequently preceded by Tau Ser422 phosphorylation.^50^ In our hands, JNK3 overexpression was enough to induce all the aberrant conformations studied not only in Tg2576 but also in WT mice, suggesting that Tau misfolding and subsequent microtubule disaggregation could be also underlying the cognitive deficiencies observed in AAV-JNK3 mice.

In conclusion, in the present work, we show that pJNK expression is significantly increased in AD, while similar pJNK expression was found in other dementias. Furthermore, there was a significant correlation, co-localization, and direct interaction between pJNK expression and Aβ levels in AD. Significant increased levels of pJNK were also found in Tg2576 mice, a model of AD. Moreover, JNK3 overexpression, achieved by intrahippocampal injection of an adeno-associated viral vector expressing this protein, was enough to induce cognitive deficiencies and precipitate Tau aberrant misfolding in Tg2576 mice without accelerating amyloid pathology.

Altogether, we can propose that AD characteristic amyloid pathology could lead to pJNK increase and activation, which in turn could induce neuroinflammation and Tau misfolding, inducing a vicious cycle that could lead to cognitive deficiencies and neurodegeneration.

## Methods

### Cells

BHK-21 cells (ATCC: CCL-10) and derived stable cell lines were cultured in BHK-21 Glasgow MEM (Gibco BRL, UK) supplemented with 5% FCS, 10% tryptose phosphate broth, 2 mM glutamine, 20 mM HEPES, 100 μg/mL streptomycin, and 100 IU/mL penicillin (BHK complete medium). HEK-293T (ATCC CRL-3216) cells were grown in DMEM (Gibco BRL) supplemented with 10% FBS, 2 mM glutamine, 100 μg/mL streptomycin, and 100 U/mL penicillin.

### Patients, Clinical and Neuropathological Data, and Tissue Processing

Frontal (Brodmann area, BA10) cortices were obtained from the Brains for Dementia Research Initiative Network (BDR). At death, informed consent had been obtained from the patients’ next of kin before collection of brains. AD cases were clinically diagnosed on the basis of meeting the Consortium to Establish a Registry for Alzheimer’s Disease (CERAD) criteria,^51,52^ Lewy body dementia (LBD) according to international consensus criteria,^53^ and frontotemporal dementia (FTD) according to Movement Disorders Society criteria.^54^ Vascular dementia (VaD) was defined by the presence of multiple or cystic infarcts. All tissue used had a brain pH > 6.1, the condition used as an indication of tissue quality in postmortem research.

### Animals

Nine and 16 month-old Tg2576 AD transgenic and wild-type mice were used (*n* = 7–10). Intracerebroventricular (ICV) injection of Aβ was performed in 9 month-old wild-type C57BL/6J mice (*n* = 14).

Animals were housed in a temperature (21 ± 1 °C)- and humidity (55 ± 1%)-controlled room on a 12 h light/dark cycle. Experimental procedures were conducted in accordance with the European and Spanish regulations (2003/65/EC; 1201/2005) for the care and use of laboratory animals and approved by the Ethical Committee of University of Navarra (ethical protocol numbers 068-11 and 038-17).

### Aβ Intracerebroventricular Injection

The Aβ_42_ peptide (Bachem Laboratories) was oligomerized as described in ref (55). ICV injection of Aβ_42_ (1 μL in sterile PBS) was stereotaxically performed in both lateral ventricles (anterior–posterior, +0.3 mm; lateral, 1.0 mm; horizontal, 3.0 mm from the bregma). Sham animals received equivalent amounts of sterile PBS. Mice were sacrificed 7 days after the injection.

### Plasmid

A synthetic gene containing the coding sequences of mouse JNK3 isoform (NCBI Reference Sequence: NP_001075036.1) and of green fluorescent protein (GFP) bound by the IRES (internal ribosome binding site) sequence of the encephalomyocarditis virus was generated in the company GenScript (Piscataway, USA). The synthetic cassette was subcloned into the pAAV-CAG-GFP plasmid, substituting the GFP gene, generating the pAAV-CAG-JNK3-GFP plasmid.^56^

### Viral Vector Production

Recombinant single-stranded AAV8 vectors were purified from HEK-293T cells that had been co-transfected using 25 kDa linear polyethylenimine (Polysciences, Warrington, PA, USA) with two different plasmids: a plasmid containing ITR-flanked transgene constructs (pAAV-CAG-JNK-GFP) and a plasmid containing the adenoviral helper genes and AAV8 cap & rep genes (named pDP8.ape; Plasmid Factory, Bielefeld, Germany). Seventy-two hours post transfection, the supernatant was collected and treated with polyethylene glycol solution (PEG8000, 8% v/v final concentration) for 48–72 h at 4 °C. The supernatant was then centrifuged at 1500*g* for 15 min. Cells containing AAV particles were collected and treated with lysis buffer (50 mM Tris–Cl, 150 mM NaCl, 2 mM MgCl_2_, and 0.1% Triton X-100) and kept at −80 °C. Three cycles of freezing and thawing were applied to both the supernatant and cell lysate. Viral particles obtained from the cell supernatant and lysate were purified by ultracentrifugation at 350,000*g* for 2.5 h in a 15–57% iodixanol gradient.^57^ The viral batches were then concentrated further by passage through Centricon tubes (YM-100; Millipore). All vector stocks were kept at −80 °C until use.

AAV vector titers (viral particles (vp)/mL) were determined by quantitative PCR for viral genome copies extracted from DNAase-treated viral particles (High Pure Viral Nucleic Acid Kit, Roche). The primers used in q-PCR were Forward-eGFP (5′-GTCCGCCCTGAGCAAACA-3′) and Reverse-eGFP (5′-TCCAGCAGGACCATGTGATC-3′). Vector titers obtained were >10^12^ viral genomes (VG)/mL.

### Analysis of JNK3 Expression *In Vitro*

BHK cells were transfected with 2, 4, and 6 μg of pAAV-CAG-JNK3-GFP plasmids using Lipofectamine 2000 (Thermo Fisher). Cells were fixed at 24 and 48 h, and pJNK and total JNK expression was detected by immunoblotting and immunofluorescence using a primary mouse monoclonal antibody specific for anti-pJNK and anti-total JNK (both 1:1000, Cell Signaling). A donkey anti-rabbit Alexa-546-conjugated antiserum (Invitrogen, 1:1000) was used for detection.

### Intrahippocampal Injection

Intrahippocampal injection of AAV-JNK3 (1 × 10^10^ VG) was performed in 6 month-old Tg2576 AD transgenic mice (*n* = 6 per group) stereotaxically in both hemispheres, with the following coordinates: anterior–posterior, −2 mm; medial–lateral, ±1.4 mm; dorso-ventral, −1.8 mm from the bregma. Sham animals (*n* = 12) received equivalent amounts of sterile PBS. Behavioral test and sacrifice were performed 3 months after the injection.

### Behavioral Test

Behavioral experiments were conducted between 09:00 and 13:00 h. Observers were blind to the genotype. All tests were carried out using a video-tracking system (Ethovision 3.0; Noldus Information Technology BV).

#### Open Field

Locomotor activity was measured for 30 min in an open field (35 × 35 cm, 45 cm height) in a softly illuminated room. The total path (cm) was analyzed.

#### Novel Object Recognition Test (NORT)

The open field consisted of a square divided into four sections (35 cm × 35 cm × 45 cm each). On the previous day to the experiment, animals were familiarized with the square for 30 min. The test consists of three trials of 5 min: sample phase, 1 h trial, and 24 h trial. During the first trial, two identical objects were placed inside the cubicle, and the mice were allowed to explore. One or 24 hours later, the second task took place in which one object was replaced by another and the exploration time was recorded for 5 min. Results were expressed as the percentage of time spent exploring the new object with respect to the total exploration time (discrimination index).

#### Morris Water Maze (MWM)

The water maze is a circular pool (diameter of 145 cm) filled with water (21–22 °C) and virtually divided into four equal quadrants identified as northeast, northwest, southeast, and southwest.

To test the learning capacity, hidden-platform training was conducted with the platform placed in the northeast quadrant 1 cm below the water surface over 9 consecutive days (four trials/day). Several large visual cues were placed in the room to guide the mice to the hidden platform. Each trial was finished when the mouse reached the platform (escape latency) or after 60 s, whichever came first. Mice failing to reach the platform were guided onto it. After each trial, mice remained on the platform for 15 s. To test memory, probe trials were performed at the 4th, 7th, and last day of the test (10th day). In the probe trials, the platform was removed from the pool and mice were allowed to swim for 60 s. The percentage of time spent in the target quadrant was recorded.

### Tissue Collection

Mice were sacrificed by decapitation. Brains were removed and dissected on ice to obtain the hippocampus and frontal cortex and stored at −80 °C. For immunohistochemistry assays, left hemispheres from five mice per group were fixed by immersion in 4% paraformaldehyde in 0.1 M PBS (pH = 7.4) for 24 h, followed by 30% sucrose solution. Brains were cut into a series of 40 μm slides.

### Western Blotting

BA10 from patients and the frontal cortex or hippocampus of mice were homogenized in ice-cold RIPA buffer and centrifuged at 13,000*g* and 4 °C for 20 min, and the supernatant samples were separated on 7.5% polyacrylamide gels. The primary antibodies used were pJNK (1:1000, Cell Signaling), total JNK (1:1000, Cell Signaling), Tau MC1 epitope and Tau ALZ50 epitope (both 1:1000, donated by Peter Davies, Albert Einstein College of Medicine), Asp421-cleaved Tau clone C3 (1:1000, Merck), and Ser422 phospho-Tau (1:1000, Thermo Fisher). Secondary antibodies conjugated to IRDye 800CW or IRDye 680CW (LI-COR Biosciences) were diluted to 1:5000 in TBS with 5% BSA. Bands were visualized using the Odyssey Infrared Imaging System (LI-COR Biosciences). β-Actin (1:10000, Sigma-Aldrich) was used as an internal control.

For the visualization of Aβ oligomers, tissue was homogenized, divided by ultracentrifugation (100,000*g*, 1 h, 4 °C), and subjected to SDS-PAGE electrophoresis in 12% gels and nonthermally denaturated conditions (samples were not boiled before loading). The separated proteins were transferred to nitrocellulose membranes for determining the presence of different Aβ aggregates with 6E10 as the primary antibody (1:1000, Covance).

### Measurement of Aβ Levels

Aβ42 levels were measured using a commercially available ultrasensitive ELISA kit (Thermo Fisher Scientific) following the manufacturer’s instructions.

### Immunofluorescence Staining

For immunofluorescence, free-floating brain sections were washed (3 × 10 min) with 0.1 M PBS (pH = 7.4) and incubated in blocking solution (PBS containing 0.3% Triton X-100, 0.1% BSA, and 2% normal donkey serum) for 2 h at room temperature. For 6E10 immunostaining, sections were incubated in 70% formic acid for 10 min before blocking. Sections were incubated with the primary antibody overnight at 4 °C, washed with PBS, and incubated with the secondary antibody for 2 h at room temperature, protected from light. The primary antibodies used were anti-pJNK (1:250, Cell Signaling), 6E10 (1:250, Covance), anti-GFAP (1:1000, Cell Signaling), anti-NeuN (1:1000, Cell Signaling), AT8 antibody (1:1000, Cell Signaling), and anti-GFP (1:1000, Invitrogen). Secondary antibodies used were Alexa Fluor 488 Donkey anti-rabbit IgG and Alexa Fluor 546 Donkey anti-mouse IgG (1:200, Invitrogen-Molecular Probes).

To develop the human brain section immunofluorescence, slides were dewaxed and washed with xylol, decreasing concentrations of ethanol (100, 90, and 70%), and water during 5 min every time. Sections were washed with 3% hydrogen peroxide during 5 min at 37 °C and then immersed in dH_2_O (2 × 5 min) and 0.1 M PBS (pH = 7.4; 2 × 5 min). With the purpose of antigen retrieval, slides were treated with 0.01 M citrate buffer (pH = 6) and microwaved for 2 min. After performing the staining and before mounting, Sudan black staining was applied to brain sections and they were washed with 70% ethanol for 1 min and dH_2_O (3 × 5 min).

The primary antibodies employed were anti-pJNK (1:250, Cell Signaling Technology) and 6E10 (1:250, Covance). Secondary antibodies used were Alexa Fluor 488 Donkey anti-rabbit IgG and Alexa Fluor 546 donkey anti-mouse IgG (1:250, Invitrogen-Molecular Probes). Fluorescence signals were detected with confocal microscope LSM 510 Meta (Carl Zeiss).

### Immunohistochemistry

Immunohistochemical examination of brains was performed using mouse monoclonal antibodies against Tau MC1 epitope and Tau ALZ50 epitope (both 1:100, donated by Peter Davies), Asp421-cleaved Tau clone C3 (1:250, Merck), and Ser422 phospho-Tau (1:250, Thermo Fisher). Antibody binding was detected with a biotinylated secondary antibody, and the antibodies were visualized using an avidin–biotin–peroxidase complex with 3,3′-diaminobenzidine tetrahydrochloride (DAB) as the chromogen.

### Quantitative Reverse Transcription Polymerase Chain Reaction (qRT-PCR)

For qRT-PCR analysis, total RNA was extracted from respective tissues using TRIzol reagent. Isolated total RNA was reverse-transcribed into cDNA using commercially available kits (Applied Biosystems). All subsequent qRT-PCR reactions were performed on a QuantStudio 7 Flex Real-Time PCR System (Applied Biosystems). For normalization, all replicate analyses were normalized to GAPDH. The following Taqman probes (Applied Biosystems) were used: MAPK10 (Mm00436518_m1) and GFP (Mr03989638_mr).

### Proximity Ligation Assay (PLA)

These experiments were carried out using the Duolink In Situ Red PLA detection kit (DUO92101, Sigma) according to the manufacturer’s protocol and using 6E10 and JNK primary antibodies.^58,59^

### Statistical Analysis

Results, reported as means ± SEM, were analyzed by GraphPad Prism 6.0, and normality was checked by Shapiro–Wilk’s test (*p* < 0.05). In the acquisition phase of the MWM, overall treatment effects were examined by two-way repeated measure ANOVA (treatment × trial). Data with two variables (genotype × AAV) were analyzed with two-way ANOVA followed by Tukey test. Data with more than two independent variables were analyzed with one-way ANOVA. In all cases, the significance level was set at *p* < 0.05.

## Supporting Information Available

Table describing the demographic features of patients (Table S1), correlation between pJNK levels and the age of control subjects (Figure S1), pJNK and brain cell co-localization in AD brains (Figure S2), and JNK3 expression *in vitro* (Figure S3) (PDF)
