# Supplementary material for: JNK Activation in Alzheimer’s Disease Is Driven by Amyloid β and Is Associated with Tau Pathology
Source: ACS Chem Neurosci. 2023 Mar 28;14(8):1524–34. doi: 10.1021/acschemneuro.3c00093 (PMC10119940; doi:10.1021/acschemneuro.3c00093)
Supplement: Supplementary file 1 — cn3c00093_si_001.pdf [file cn3c00093_si_001.pdf]

Supporting Information

JNK activation in Alzheimer's disease is driven by amyloid β and is associated with Tau pathology

Maite Solas, PhD[1,2*], Silvia Vela, PhD[1], Cristian Smerdou, PhD[2,3], Eva Martisova, PhD[3], Iván Martínez-Valbuena, PhD[2,4,5], María-Rosario Luquin, PhD[2,4,6], María J. Ramírez, PhD[1,2]

[1]Department of Pharmacology and Toxicology, University of Navarra, 31008 Pamplona, Spain; [2]IdISNA, Navarra Institute for Health Research, 31008 Pamplona, Spain; [3]Division of Gene Therapy and Regulation of Gene Expression, Cima Universidad de Navarra, 31008 Pamplona, Spain; [4]Neurosciences Division, Cima Universidad de Navarra, 31008 Pamplona, Spain; [5]Tanz Centre for Research in Neurodegenerative Diseases, University of Toronto, M5S 1A8 Toronto, Canada; [6]Neurology Department, Clinica Universidad de Navarra, 31008 Pamplona , Spain.

*Correspondence: msolaszu@unav.es (Maite Solas)

Department of Pharmacology and Toxicology

School of Pharmacy

University of Navarra

Irunlarrea, 1

31011 Pamplona (Spain)

+34 948425600 (806648)

Table 1. Demographic features of patients

Diagnosis	Gender (male/female)	Age at death (years)
Controls		
Mature (n=10)	5/5	65.10 ± 3.86
Old (n=16)	6/10	77.14 ± 2.77
AD (n=16)	5/11	81.20 ± 1.90
FTD (n=11)	6/5	61.91 ± 2.48
LBD (n= 8)	4/4	74.50 ± 2.73
VaD (n=8)	4/4	72.12 ± 3.77

AD: Alzheimer’s disease. FTD: Frontotemporal dementia. VaD: Vascular dementia. LBD: Lewy Body dementia.

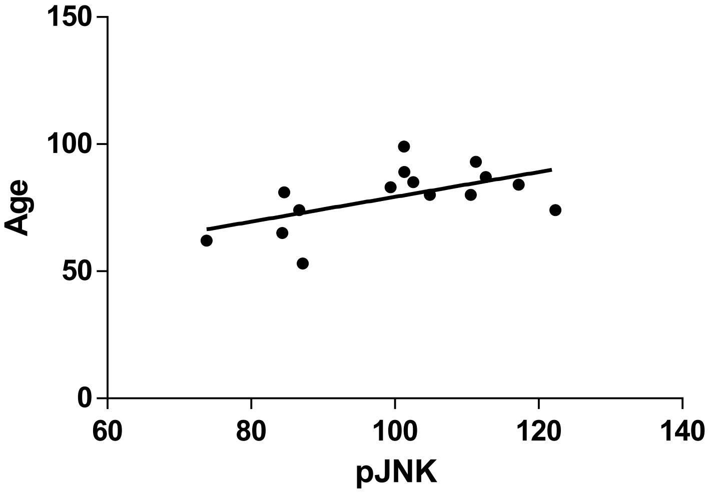

Fig. S1: Correlation between pJNK and age in control subjects, showing an age dependent pJNK increase in human brain samples. Pearson's, r=0.563; p<0.05.

Supplementary Figure 1

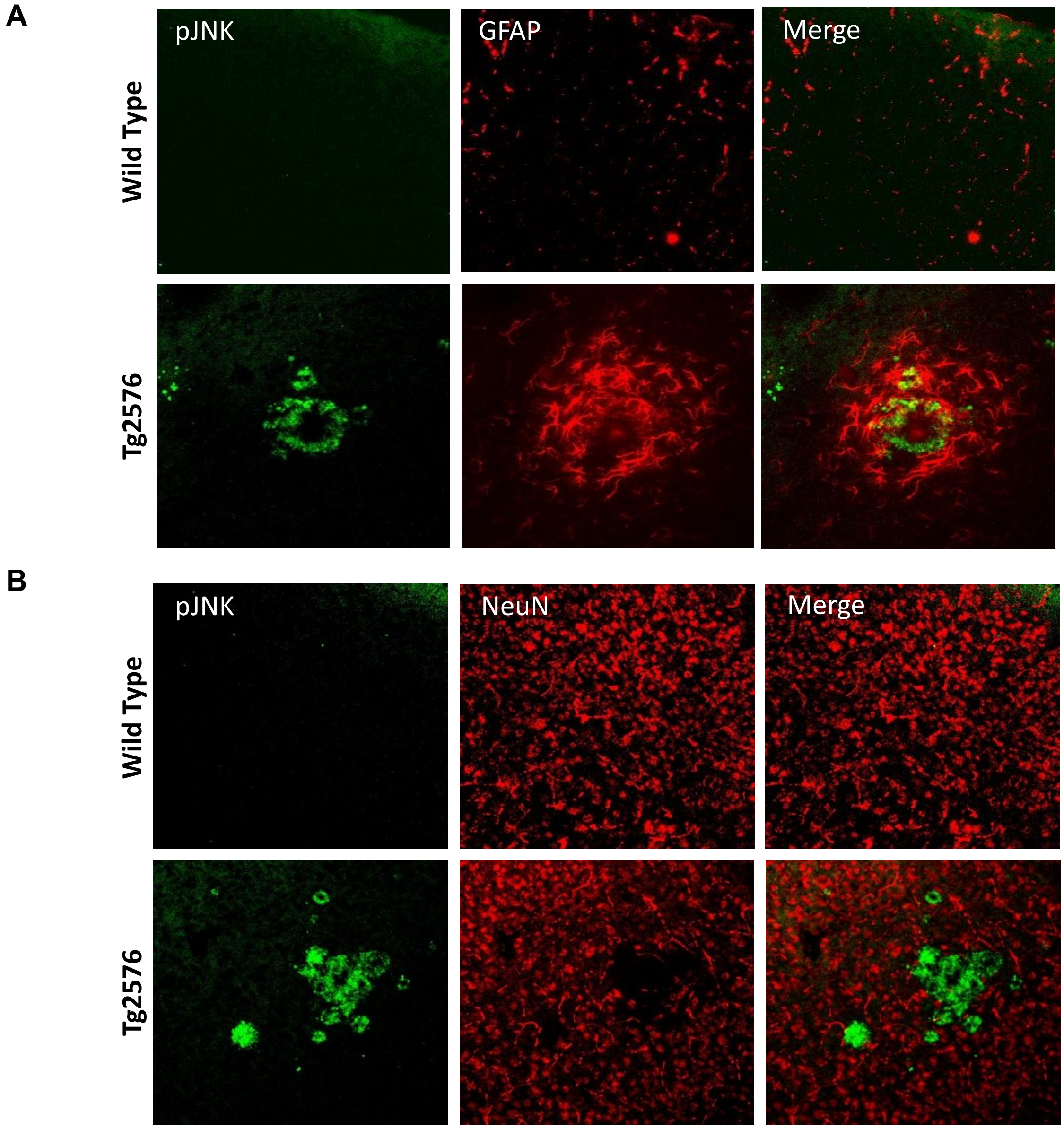

Fig. S2: pJNK and brain cells co-localization in AD brains. A pJNK and GFAP (astrocytic marker) and **B** pJNK and NeuN (neuronal marker) immunostaining in in frontal cortex of Tg2576 mice. Scale bar 50 µm.

Supplementary Figure 2

A

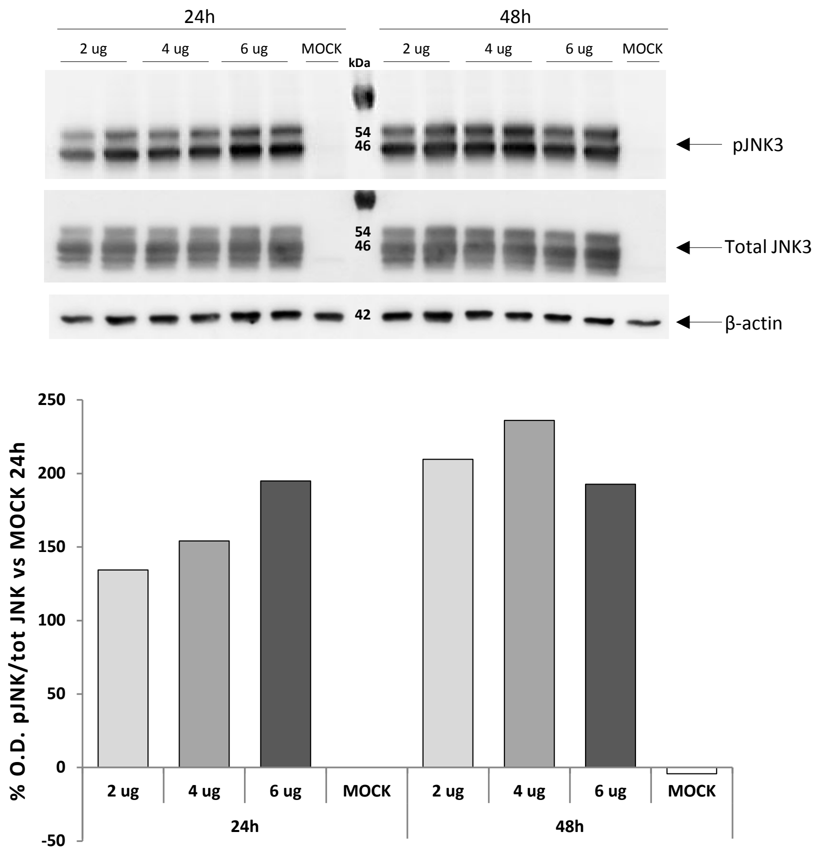

B

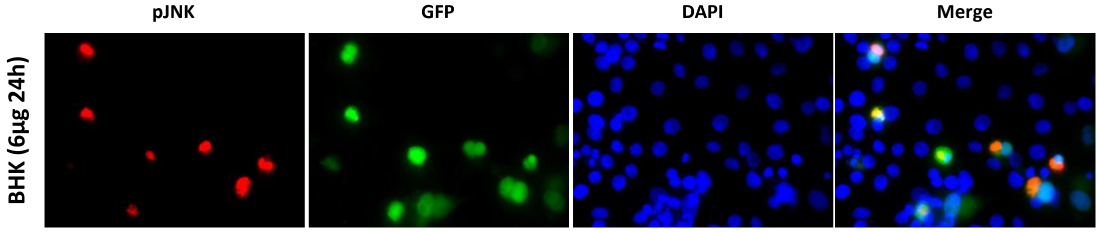

Fig. S3: JNK3 expression *in vitro*. BHK cells were transfected with 2, 4, and 6 µg of pAAV-CAG-JNK3-GFP plasmids and analyzed at 24 h and 48 h by **A** immunoblotting and **B** immunofluorescence with an antibody specific against JNK3 (detected in red to allow colocalization with GFP expressed form the same vector). Results are shown as mean ± SEM. In panel A figures show optical density (O.D.) values percentage and an illustrative image of the blotting.
